# Medico-Chirurgical Society of Edinburg

**Published:** 1844-09-07

**Authors:** 


### MEDICO-CHIRURGICAL SOCIETY OF EDINBURG.

### SCIRRHUS OF THE UTERUS.

Dr. Scott read a case of scirrhus of the uterus in which none of the usual signs of that disease had appeared, but which, two years previous to death, presented all the symptoms of spinal neuralgia, constant cough without any indication of disease of the lungs, vomiting of food, exquisite pain almost to fainting on pressure of the spine, and progressive emaciation.

The disease terminated fatally from slight uterine hæmorrhage. On examination, the lungs and stomach were found healthy; the uterus was schirrous and enlarged, but without ulceration. Dr. Scott considered it, in connection with the papers he had formerly communicated to the Society, as throwing great light on the subject of spinal neuralgia in general.

### ABDOMINAL HYDATIDS.

Dr. Gairdner, read a remarkable case of abdominal disease, in which he had twice withdrawn by paracentesis from the peritoneal cavity, a quantity of a glutinous substance, resembling in colour and consistence, calf's foot jelly, and coagulating, like the albumen of eggs, by heat. Some of this matter Dr. G. had exhibited to the society in January, but in a comminuted state, in consequence of having passed through the canula, and the valves of the syringe adapted to it for suction. After the death of the patient in February last, it was ascertained that the whole abdomen was filled with similar matter, amounting in all to about twenty-four imperial pints, and that it consisted of rounded masses having very much the same outward appearance as the ordinary acephalocysts of Laennec, but differing from them in many important particulars. These remarkable parasites had invaded the textures of the liver and omentum, as well as some of the other viscera. The right lobe of the liver was entirely destroyed by them; the left partially; the omentum converted into a dense and almost cartilaginous tumour, of large size and great thickness, in which many of the parasites were contained, and from which many of them hung pendulous. Dr. G., after detailing the facts of the case, gave a condensed account of the results of his inquiries into the literature of the subject. He found that extensive destruction of the liver by hydatids, was by no means uncommon, and that there were one or two recorded cases, and more especially a very remarkable one by Ruysch, in which the destruction of this organ appeared to have been equally extensive as in his own case. He had not been successful in finding any case in which the omentum had undergone a change of the same sort precisely with that which he had described. Finally, he had examined attentively all the systematic treatises on entozoa in general, or on hydatids in particular, which were accessible to him, besides a great number of pathological works and individual cases in which they were incidentally described, and he had not yet found a single description of a parasite possessing the same physiological and structural peculiarities. He was therefore of opinion, that it was a rare one; and that, although it had in all probability been occasionally seen, its peculiarities had not yet been observed or described.

STRUCTURAL AND PHYSIOLOGICAL PECULIARITIES OF THE ABOVE ENTOZOON.

Mr. Goodsir read to the Society an account of the structural and physiological peculiarities of the entozoon found in Dr. Gairdner's case, by Mr. Henry D. S. Goodsir. Mr. Goodsir, after quoting from his brother's paper, read to the Royal Society, a description of the animal in question, explaining more particularly the structure of the parasite—stated as its most distinctive peculiarity, the fact, that instead of being like the common acephalocyst, a simple animal, it might be regarded as being of a composite nature. It appeared to consist of several cells having in their interior a glutinous matter contained in cellular tissue, connected together by a common membrane extending from the free surface of the peritoneum over the surface of the various cysts, and forming their pedicles. On the surface of this membrane, except on the part covering the globular cysts, there were seen numerous circular discs, around the margin of which were a series of stomata opening into tubes extending into the substance of the membrane. These Mr. Henry Goodsir regarded as being the nutritive organs of the animal. With regard to its reproduction, Mr. Goodsir remarked, that this took place in two ways, one which respected the extension of the individual existing group, the other as regarded the propagation of the animal to uninfected tissues. This, like the common hydatid, did not enlarge from cellular development, but by simple expansion of the original germinal vesicle. As regards its propagation to uninfected tissues, Mr. Goodsir stated, that he had been unable to trace the earlier process by which an ovum appeared in the healthy tissue, but he found that when this had occurred, the ovum enlarging makes its way like an abscess to the free surface of the organ, and bursts, leaving an orifice leading into a cavity of the tissue. The formation of these cavities gave to the peritoneum the honey-comb appearance, observable in Dr. Gairdner's preparations. After the bursting of the sac, the animal is not discharged from the cavity, but remains attached by its common membrane to the base of the cavity, and then projects outwards elongating its outer membrane so as to form a pedicle.

CAROTID ANEURISM.

Dr. Duncan read the case of a woman aged 33, in whom he had tied the carotid artery for aneurism. The aneurism was of very large size. On her admission into the hospital it extended from the angle of the inferior maxilla to about two fingers' breadth from the clavicle. Two or three days after her admission she was seized with spasmodic attacks of dyspnœa, one of which was followed by a state of complete insensibility, the pulse being almost imperceptible, and the respirations only five in the minute. Tracheotomy was performed by Dr. D.; and on her rallying, it being apprehended that the sac of the aneurism might give way, the vessel was tied. Everything promised well until the 12th day, when inflammation of the sac of the aneurism followed, and a small ulcerated communication with the pharynx formed. The sac was then freely incised, and the coagula turned out, but the woman died on the 15th day. No morbid appearnces sufficient to account for death were found at the post mortem inspection; and it was believed that the cause of death was spasm of the glottis, induced by irritation of the recurrent nerve, which was involved in the tumour.

ON THE SOURCES OF REPRODUCTION AFTER THE DEATH OF THE SHAFT OF A LONG BONE.

Mr. Goodsir remarked, that bone, as a tissue, was to be regarded as an extra-vascular osseous matter, accompanied by its membranes, which are three in nnmber—the periosteum, the medullary membrane, and the membrane lining the Haversian canals. As it is to this last membrane that the original formation of osseous tissue is owing, so, to morbid actions in it, can be traced many of the pathological conditions, such as inflammation of bone, ulceration of bone, &c. As to the sources from which osseous tissue is derived after the death of the shaft of a long bone, Mr. Goodsir observed, that there were, 1*st.* portions of the old shaft removed by ulceration previous to its death, and 2*d*, portions of the membrane of the Haversian canals remaining attached to the periosteum. Mr. Goodsir did not believe that any new bone was formed from the periosteum, properly so called, or from the epiphyses, as has been supposed by some pathologists.—*Lond. & Ed. Monthly Jour.*

